# How Can Wake-up Radio Reduce LoRa Downlink Latency for Energy Harvesting Sensor Nodes?

**DOI:** 10.3390/s21030733

**Published:** 2021-01-22

**Authors:** Nour El Hoda Djidi, Matthieu Gautier, Antoine Courtay, Olivier Berder, Michele Magno

**Affiliations:** 1 Institut de Recherche en Informatique et Systèmes Aléatoires (IRISA), National Centre for Scientific Research (CNRS), University Rennes, 22300 Lannion, France; matthieu.gautier@irisa.fr (M.G.); antoine.courtay@irisa.fr (A.C.); olivier.berder@irisa.fr (O.B.); 2 Department of Information Technology Electrical Engineering, ETH Zürich, 8092 Zurich, Switzerland; michele.magno@pbl.ee.ethz.ch

**Keywords:** internet of things, wake-up radio, LoRa, heterogeneous networks architecture, opportunistic cluster heads, energy harvesting

## Abstract

LoRa is popular for internet of things applications as this communication technology offers both a long range and a low power consumption. However, LoRaWAN, the standard MAC protocol that uses LoRa as physical layer, has the bottleneck of a high downlink latency to achieve energy efficiency. To overcome this drawback we explore the use of wake-up radio combined with LoRa, and propose an adequate MAC protocol that takes profit of both these heterogeneous and complementary technologies. This protocol allows an opportunistic selection of a cluster head that forwards commands from the gateway to the nodes in the same cluster. Furthermore, to achieve self-sustainability, sensor nodes might include an energy harvesting sub-system, for instance to scavenge energy from the light, and their quality of service can be tuned, according to their available energy. To have an effective self-sustaining LoRa system, we propose a new energy manager that allows less fluctuations of the quality of service between days and nights. Latency and energy are modeled in a hybrid manner, i.e., leveraging microbenchmarks on real hardware platforms, to explore the influence of the energy harvesting conditions on the quality of service of this heterogeneous network. It is clearly demonstrated that the cooperation of nodes within a cluster drastically reduces the latency of LoRa base station commands, e.g., by almost 90% compared to traditional LoRa scheme for a 10 nodes cluster.

## 1. Introduction

Low-Power Wide Area Networks (LPWANs) have gained in the recent years a significant interest by both research community and industry for different Internet of Things (IoT) applications, such as smart cities, smart health, and smart farms. Such technology offers a link range of several kilometers and a low power consumption, but at the cost of small data rate. Many technologies belong to LPWAN category, such as SigFox, Narrowband IoT, Weightless, and LoRa [1]. Among these existing LPWANs sets, LoRa is the most popular due to its open communication protocol (LoRaWAN) and its ability to achieve a long range and to recover data from weak signals even below the noise floor [2]. Therefore, many projects were conducted with LoRa technology in different applications, such as in smart farms, where animal health monitoring and tracking is having a growing interest [3]. Three classes are defined in the LoRaWAN specification (A, B, and C) that will be detailed in Section 2.1. A tradeoff needs to be made between power consumption and downlink latency for these three classes.

Wake-up Radio (WuR) is a consolidated technology that helps to achieve the trade-off between power consumption and latency. WuR is a secondary always-on Ultra Low Power (ULP) radio subsystem that is connected to the main node. The WuR power consumption is several orders of magnitude less than that of the main node, but it has a short range communication capability [4]. The WuR is continuously listening to the channel while the main radio is in sleep mode, and, when a specific signal called Wake-Up Beacon (WUB) is received, the WuR wakes up the main radio through an interrupt. Thus, the WuR allows an asynchronous wake-up of the main node with a low latency. Recent circuit designs of WuR embed a decoding capability through a ULP-MCU or a correlator allowing to wake up only a specific node, thus reducing considerably the waste of energy consumption of the main radio [5].

As LoRa and WuRs present orthogonal features (long range with LoRa and short range with WuRs), recent works have proposed to combine these two technologies to achieve an energy efficiency and reduce the downlink latency [6,7]. Nevertheless, LoRa resilience is limited if the devices are battery powered, which also limits their deployment in constrained environments as the cost of the battery replacement is high in difficult to access environments. Therefore, improving energy efficiency is not sufficient in itself. Energy harvesting, that converts energy from environmental sources, is a viable alternative to ensure sustainable operation and perpetually power devices or at least extend their lifetime [8].

This work investigates the use of energy harvesting, in a network architecture that combines the two technologies LoRa and WuR, with an adequate MAC protocol LoRa-WuR to reduce the downlink latency and to keep nodes self sustainable. Another challenge is to achieve a consistent downlink Quality of Service (QoS) (i.e., the received command rate) with low fluctuation between periods of plenty energy and sparse energy. To that goal, the node uplink QoS (i.e., the packet generation rate) is tuned according to the harvested energy by a software component called energy manager. In this work, a novel energy manager dedicated to LoRa-WuR MAC protocol is presented, allowing an improvement of both downlink latency and downlink QoS consistency. Therefore, the main contributions of this work are:

- A novel energy manager suitable to heterogeneous nodes with energy harvesting capabilities.
- A new MAC protocol leveraging WuR that reduces LoRaWAN downlink latency.
- Hybrid energy and latency models based on real platform microbenchmarks.
- Evaluation of the influence of energy harvesting conditions on the downlink QoS and the latency.

This paper is structured as follows. In Section 2, related works are given. Section 3 details the network architecture and the MAC protocol. A latency and average power consumption models and evaluation with experimental measurements are presented in Section 4. A use case with energy harvesting nodes is proposed in Section 5, followed by a conclusion in Section 6.

## 2. Related Works

The related works concern both long range and WuR communication technologies and the combination of these two technologies in a heterogeneous architecture. The energy harvesting dedicated to LoRa sensor nodes is also investigated.

### 2.1. Long Range Communications

Some IoT applications require both long range connectivity and low power consumption, and recent LoRa technology allows such performance. LoRa physical layer uses the Chirp Spread Spectrum (CSS) modulation which allows a resilient communication to interference and a long coverage with low power characteristics [9,10]. Different configuration parameters can be exploited with LoRa: the carrier frequency, the spreading factor SF, the bandwidth BW, and the coding rate CR. The combination of these parameters provides different tradeoffs between battery lifetime and transmission range [11]. The bit-rate $R_{b}$ is calculated by:

(1) $$R_{b}=SF\frac{BW}{2^{SF}}CR.$$

SF represents the number of chips per symbol and can take values from 6 to 12. The higher the value is, the more time is taken to send a packet and the higher range is achieved. The coding rate CR can take the following values: $\frac{4}{5}$, $\frac{2}{3}$, $\frac{4}{7}$, $\frac{1}{2}$. The smaller the coding rate is, the higher the time on air is and the more reliable the data transmission is. BW can be chosen among three options: 125 kHz, 250 kHz, or 500 kHz. For long range, the lowest value should be configured.

LoRa can be associated with any MAC protocol, but LoRaWAN (developed by LoRa Alliance) is currently the only standardized MAC. LoRaWAN networks typically use a star topology in which gateways gather messages from nodes, also called End-Devices (EDs), and a central network server at the backend [12]. LoRaWAN specification defines three classes:

- Class A: in this class, EDs can initiate an uplink transmission based on their own communication needs. Each uplink transmission is followed by two short downlink receive windows [13]. This class has the lowest power consumption of the ED but the highest downlink latency from the server.
- Class B: in addition to class A receive windows, EDs in class B open extra receive windows, called ping slots [14]. If no preamble from the gateway is detected during this ping slot, the ED immediately returns back to sleep. If a preamble is detected, the radio transceiver stays on until the frame is demodulated. The gateway also provides a time reference to the EDs by periodically broadcasting beacon.
- Class C: EDs in class C continuously open receive windows, only closed for transmission. These EDs consume more power than with class A or class B but offer the lowest downlink latency.

A trade-off needs to be made between downlink latency and power consumption of these three classes. Usually, in applications, such as smart building or environment monitoring, the EDs use LoRaWAN class A as it offers the lowest power consumption but at the cost of a high downlink latency. On the other hand, gateways are using class C as they are not energy constrained. Some applications, such as applications based on event driven methods, in which the gateway has a critical command to send to the EDs, require a low downlink latency. This is why we investigate the use of the WuR with LoRa.

### 2.2. Wake-up Radio

WuR is a ULP receiver that is continuously listening to the channel while consuming a few nanowatts or microwatts depending on the circuitry design. WuR is used in addition to the main transceiver and allows an asynchronous wake-up of the main node with low latency. Using the WuR allows to reduce the power consumption of the network, without suffering the bloated latency of all mechanisms of time synchronization induced by duty cycled MAC protocols [15]. When a WUB is received by the WuR, the latter wakes up the main node via an interrupt. The WUB can contain the address of the destination node to only wake-up a specific node. In such case, an address matching is performed with a ULP-MCU or a correlator. Different circuit designs of the WuR can be found in the literature, and most of them work with OOK (On-Off Keying) modulated signals [16], allowing a simplified WuR circuitry. As a consequence, WuRs have both a low sensitivity and a low bit-rate. In this work, the WuR proposed in Reference [5] is used. This WuR consumes 1.80μW in sleep mode when listening to the channel. It works at 868MHz, and achieves a sensitivity of −55 dBm. Moreover, this WuR design provides address decoding capabilities with a ULP-MCU (a PIC12LF1552 from Microchip), at a cost of few additional micro-watts when a signal is received, achieving a power consumption of 284μW.

Designing dedicated MAC protocols is important to deal with WuR features and to improve the performance of the sensor nodes. Therefore, several cross layer protocols leveraging WuR were proposed in the literature. Mahlknecht et al. proposed in Reference [17] WuR-MAC for multi-hop wireless sensor networks that allows a low power consumption and a low hop-to-hop delay thanks to the WuR. Sampayo et al. proposed in Reference [18] a MAC protocol that allows a load balancing parent selection for RPL using WuR. Djidi et al. proposed in Reference [19] WARP, a MAC protocol, that allows an adaptive power transmission and a relaying technique that improves the network lifetime. WuR was also combined with long range communications, and specific MAC protocols will be presented in the next Section.

### 2.3. Heterogeneous Architecture

Nodes can embed different radio technologies and can therefore exploit such radio diversity in order to reduce both energy consumption and latency. If it could be expected that using more than one radio technology increases the power consumption, exploiting low power radio to save the energy of high power radio can significantly reduce the global power consumption of the network. Ait Aoudia et al. proposed in Reference [6] to combine LoRa communication with WuR-based short range communication to reduce both power consumption and LoRa downlink latency. Piyare et al. proposed in Reference [20] to use the same heterogeneous architecture but with a new MAC protocol. Both of them considered a star network topology with a Cluster Head (CH) that uses LoRa in class C, which manages the received messages, from the gateway, intended to other EDs that are in the same cluster. The drawback of this architecture is the use of the CH in class C that has a high power consumption, thus inducing a short lifetime of the network. We propose in this work to extend the previous work of Djidi et al. [7] that introduced a novel MAC protocol for heterogeneous architectures combining LoRa and WuR. The novel MAC protocol allows opportunistic CH mechanism, since all EDs operate in LoRa class A, reducing significantly both downlink latency and the power consumption. In this work, we also investigate the use of energy harvesting for sensor nodes that are using this heterogeneous architecture.

### 2.4. Energy Harvesting Dedicated to LoRa Sensor Nodes

Maintaining nodes sustainably alive is important for a large deployment of IoT nodes, and improving the MAC protocol and reducing circuit power consumption are not always sufficient. Therefore, recent works are investigating the use of energy harvesting nodes in LoRa networks. Ferrero et al. investigated in Reference [21] solar, thermal, and piezo harvesting techniques for autonomous sensing applications that communicate with LoRa. Lee presented in Reference [22] a novel floating device with multi-sources energy harvesting techniques that harvests solar and thermoelectric energy and communicates with LoRa. Sherazi et al. presented in Reference [23] a LoRaWAN architecture in which the gateway is powered by an energy harvesting source. Soledad et al. proposed in Reference [24] a testbed for smart farming applications that uses LoRa technology to communicate and uses a solar panel to extend the device lifetime. Benkhelifa el al. studied in Reference [1] the resource allocation in LoRa networks supplied by ambient energy harvesting. Mabon et al. presented in Reference [25] a prototype of an energy harvesting LoRa platform with an accurate sizing of both the solar cell and the battery.

In this work, we propose to exploit the use of solar energy harvesting in the heterogeneous network architecture that combines LoRa and WuR in order to make nodes sustainable and to better explore the uplink/downlink QoS of sensor nodes and highlight the advantage of the novel MAC protocol, namely LoRa-WuR.

## 3. Architecture and MAC Protocol Design

In this section, the heterogeneous network architecture that combines LoRa and WuR, and the MAC protocol LoRa-WuR are presented.

### 3.1. Network Architecture

**Figure 1:**
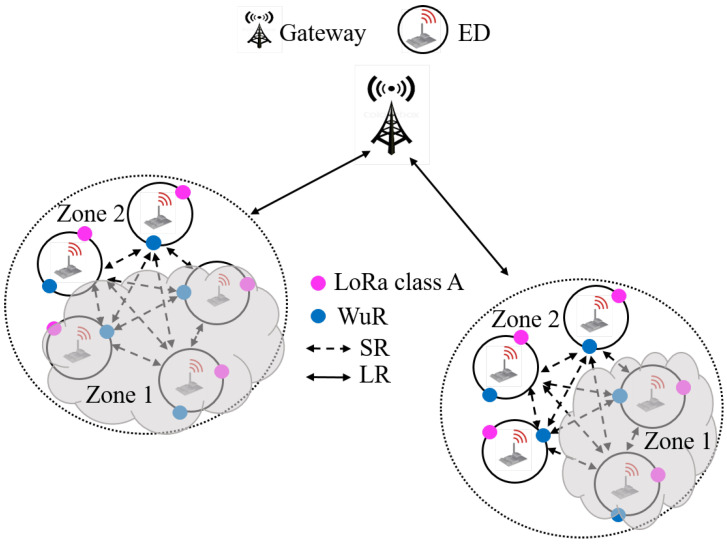
Heterogeneous network architecture with energy harvesting.

The heterogeneous network architecture considered in this work is shown in Figure 1. Nodes are distributed in clusters in which they can communicate with each other using Short Range (SR) communication [6,26]. At a distance of few kilometers from a cluster, a gateway can receive data from nodes or may send commands to the nodes using Long Range (LR) communication like the traditional LoRa scheme (i.e., LoRaWAN). The command can be, for example, a command for changing the data rate or any control command for actuators-based applications. Therefore, this network architecture targets applications for which the gateway can have control of the network for data collection, nodes configurations and even actuator activation. Each node embeds two different communication modules. The first one can handle LoRa and OOK modulations. This transceiver allows switching from both modulation schemes, LoRa is used for the LR communication with the gateway, and OOK modulation is used between nodes that are in the SR of each other. The other module is a WuR that is always listening to the channel and receives data with OOK modulation as form of a WUB. Nodes use LoRa class A to communicate with the gateway; thus, they are passing most of their time in sleep state and are only wake-up to send data to the gateway or when they receive an interrupt from the WuR. Furthermore, all nodes are equipped with an energy harvesting device, for example, a solar panel. In a real deployment, some nodes will obviously harvest less power than others, either because a problem occurs in their solar panel, or being hidden by an obstacle or being under the cloud. Therefore, two types of harvesting conditions are considered in Figure 1: nodes that harvest less energy are in zone 1 and those that harvest more energy are in zone 2. Nodes that are in zone 2 will maximize their uplink QoS that is expressed in terms of packet generation rate. On the contrary, those who are in zone 1 will reduce the uplink QoS as they harvest less energy. The uplink QoS tuning according to the harvested will be presented in Section 5.1.

### 3.2. LoRa-WuR MAC Protocol

**Figure 2:**
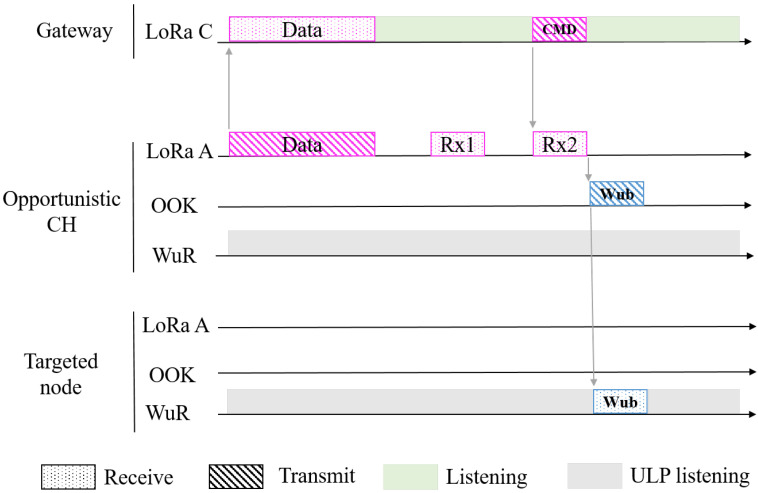
LoRa-WuR MAC protocol.

The proposed MAC protocol is described in Figure 2. It concerns nodes that are in the SR of each other, thus forming one cluster. It is assumed that the gateway already knows the location of all nodes and all the potential clusters. Nodes communicate with the gateway using LoRa class A. When a node sends data to the gateway, it opens two receive windows as it is in class A and becomes systematically an Opportunistic Cluster Head (OCH). Therefore, the gateway can take the opportunity from one of its receive windows to send Command (CMD) intended to another node called targeted node. If the OCH receives during one of its receive windows a CMD that it is intended to the targeted node, it switches its LoRa module to OOK modulation and forwards the CMD in the SR to the targeted node as form of a WUB. The WUB contains the address of the targeted node and the CMD itself. The targeted node will receive the WUB via its WuR. Thanks to this MAC protocol, namely LoRa-WuR, the downlink latency will considerably be reduced, as the gateway will take the opportunity from any receive window of any node. Nodes that are in zone 1 (low energy), having a lower uplink QoS than those in zone 2 (high energy), become less frequently OCH than those in zone 2, but all nodes cooperate together as each node can become an OCH during one of its LoRa receive window.

## 4. Latency and Power Consumption Models and Evaluation

In this section, the models of average downlink latency and average downlink power consumption of nodes using both the traditional LoRa scheme and LoRa-WuR are given. For an accurate evaluation of the models, experimental measurements from microbenchmark are included in the models. The average downlink latency is evaluated for different uplink QoSs, and the uplink QoS is tuned in Section 5.1 according to the harvested energy.

### 4.1. Latency Model

**Figure 3:**
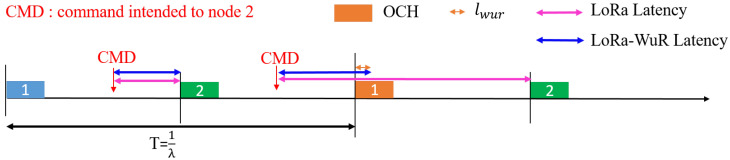
Chronogram illustrating the downlink latency.

Figure 3 illustrates the difference of latency between the traditional LoRa scheme and LoRa-WuR one. A chronogram with only two nodes is presented for clarity purpose. For LoRa scheme, when the gateway sends a CMD intended to node 2, it has to wait until the node 2 will be active to receive the CMD during its receive window. However, for LoRa-WuR scheme it has to wait any node to be active that will become an OCH, i.e., node 1 in this illustration, to transmit the CMD and then the OCH forwards it to node 2 using the SR communication.

A cluster of *N* nodes is considered and no collisions is assumed due to the low packet generation rate and the short length of WUB packets. When using LoRa class A scheme, the gateway waits for an uplink transmission of a node before sending a CMD. The average waiting time to reach a targeted node is thus $\frac{1}{2\lambda_{i}}$ [6], with $\lambda_{i}$ the packet generation rate (uplink QoS) of any node *i*. The average downlink latency $L_{LoRa}$ of the CMD transmission from the gateway to a node is expressed as:

(2) $$L_{LoRa}=\frac{1}{2\lambda_{cmd}^{LoRa}}+l_{cmd},$$

with $l_{cmd}$ the packet duration of sending the CMD using LoRa, and $\lambda_{cmd}^{LoRa}$ is a novel metric that represents the average received CMD rate by the EDs, called downlink QoS, when using the traditional LoRa scheme. As when using the traditional LoRa scheme, an ED can receive a CMD from the gateway only after its self uplink, so $\lambda_{cmd}^{LoRa}$ is equal to:

(3) $$\lambda_{cmd}^{LoRa}=\frac{\sum_{i=1}^{N}\lambda_{i}}{N},$$

with *N* the number of EDs in a cluster.

An ED that uses LoRa-WuR can receive a CMD from the gateway after an uplink of any ED that becomes an OCH; thus, the average downlink latency L${}_{LoRa-WuR}$ is expressed as:

(4) $$L_{LoRa-WuR}=\frac{1}{2\lambda_{cmd}^{LoRa-WuR}}+l_{wur}+l_{cmd},$$

with $l_{wur}$ the packet duration using the SR communication (i.e., the transmission of the WUB) which is equal to $\frac{L_{WUB}}{R_{WUB}}$, with $L_{WUB}$ the length of the WUB (bits), and $R_{WUB}$ the bit-rate of the WUB (bits/s), and $\lambda_{cmd}^{LoRa-WuR}$ the average downlink QoS when using LoRa-WuR scheme and it is expressed as:

(5) $$\lambda_{cmd}^{LoRa-WuR}=\sum_{i=1}^{N}\lambda_{i}.$$

### 4.2. Power Consumption Model

Using the LoRa class A scheme, CMDs from the gateway can only be transmitted to a node after an uplink transmission. The average power consumption of a node with an average packet generation rate λ incurred by a downlink communication denoted $P_{LoRa}$ is:

(6) $$P_{LoRa}=e_{cmd}^{L}\lambda ,$$

where *e*${}_{cmd}^{L}$ is the energy cost of receiving a CMD from the gateway using LoRa.

The average power consumption of a node incurred by the downlink communication using LoRa-WuR scheme denoted $P_{LoRa-WuR}$ is expressed as:

(7) $$P_{LoRa-WuR}=\left(e_{cmd}^{wurx}\left(N-1\right)\lambda +\left(1-\left(N-1\right)\lambda l_{wur}\right)P_{idle}^{wur}\right)+\left(e_{cmd}^{L}+e_{cmd}^{wutx}\right)\lambda ,\;N\geq 2,$$

where $e_{cmd}^{wurx}$ is the energy cost to receive and process the WUB by the WuR, $P_{idle}^{wur}$ is the power consumption of the WuR when the ULP-MCU is in a sleep state and only the radio is active listening to the channel, and $e_{cmd}^{wutx}$ is the energy cost to forward the CMD by the OCH using the SR communication.

### 4.3. Experimental Platform and Microbenchmark

**Figure 4:**
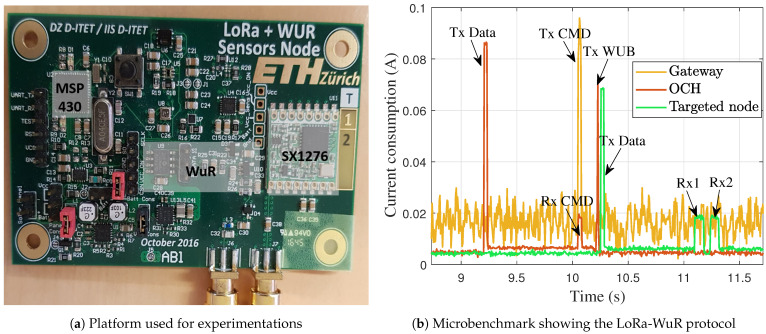
Experimental platform and microbenchmark.

The proposed LoRa-WuR MAC protocol was implemented on the platform designed in ETH Zurich [27] and shown in Figure 4a. It contains a SX1276 module from Semtech that allows OOK modulation and LoRa, a WuR from Reference [5] and an MSP430 MCU from Texas Instruments.

Figure 4b shows a microbenchmark considering three nodes, one node as a Gateway, and two EDs. It shows the current consumption of each node during a packet forwarding with LoRa-WuR protocol. The measurements were performed by using a DC power analyzer Keysight N6705B. The node (in red) that transmits the data becomes an OCH and when it receives the CMD from the gateway (in yellow), that is intended to a targeted node (in green), it forwards the CMD as form of a WUB. Once the CMD received from the OCH, the targeted node transmits data packet to the gateway using LoRa. All these steps of the protocol are illustrated in Figure 4b.

The measured power consumption, at a voltage of 3.3V, and all durations of different steps of the MAC protocol are summarized in Table 1. These measurements are used to feed the analytical models and the results are given in the next section.

### 4.4. Power Consumption and Latency Tradeoff

**Figure 5:**
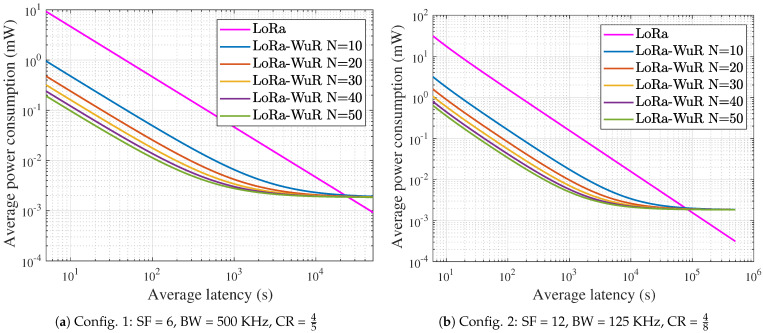
Average power consumption as a function of average downlink latency.

Figure 5 shows the average power consumption of a node as a function of the average downlink latency for both schemes LoRa and LoRa-WuR, and with different number of nodes *N* ranging from 10 to 50 in case of LoRa-WuR. Moreover, two different configurations of SF, BW, and CR are set and are listed in the Table 2, and are also noted at the bottom of both Figure 5a,b. These results are obtained from analytical models that are fed with the measured power consumption of the different operating modes. The average downlink latency and the average power consumption only depend on the downlink QoS for LoRa scheme, whereas they depend on both the downlink QoS and the number of nodes for LoRa-WuR. The uplink QoS is varied from $10^{-6}\;$ packet/s to 0.1 packet/s. It can be seen from Figure 5a that the node achieves lower power consumption and lower downlink latency than the node in Figure 5b, as it uses the lowest SF. The choice of the configuration has an impact on both downlink latency and the average power consumption of the node, but the performance gain of LoRa-WuR over LoRa scheme is about the same for both configurations. It appears from both figures that to achieve the same downlink latency with LoRa-WuR as with LoRa scheme, the average power consumption is reduced with LoRa-WuR when the downlink latency is less than 2.2$\;\times \;10^{4}\;$s and 8.3$\;\times \;10^{4}\;$s, respectively, in case of Figure 5a,b. For example, to achieve 250 s, the average power consumption is reduced down to 8.9 times (Figure 5a) and 9.4 times (Figure 5b), respectively, with 10 nodes compared to LoRa. However, when the downlink latency is very high and greater than 2.2$\;\times \;10^{4}\;$s (Figure 5a) and 8.3$\;\times \;10^{4}\;$s (Figure 5b), respectively, the traditional LoRa scheme becomes more energy efficient than LoRa-WuR because of the overhead of idle listening of the WuR. It can also be seen that, for LoRa-WuR, the higher the number of nodes is, the lower the average power consumption is. For example, to achieve a downlink latency of 250 s, the average power consumption is reduced down to 3.7 times (Figure 5a) and 4.3 times (Figure 5b), respectively, with 50 nodes compared to when using 10 nodes. This is due to the fact that, to achieve the same downlink latency, the uplink QoS needs to be higher when the number of nodes is low compared to when the number of nodes is high, which induces an increase of the power consumption.

Figure 5 also shows that the downlink latency is reduced with LoRa-WuR for a given power consumption. To achieve the same power consumption with LoRa-WuR as with LoRa scheme, the uplink QoS is reduced to compensate the overhead of forwarding the CMD by the OCH, but as nodes cooperate with each other, the downlink QoS is improved and the average downlink latency is reduced with LoRa-WuR even if the uplink QoS of a node is reduced, and the more the nodes are used in the cluster, the more reduced the downlink latency is. Therefore, the proposed LoRa-WuR protocol achieves better performance than the traditional LoRa scheme. However, this improvement is not sufficient to keep nodes sustainable. This is why the use of energy harvesting is investigated in the next section. The uplink QoS can be tuned according to the harvested energy in order to take a full advantage of the proposed MAC protocol.

## 5. Combining LoRa-WuR Architecture with Energy Harvesting

**Figure 6:**
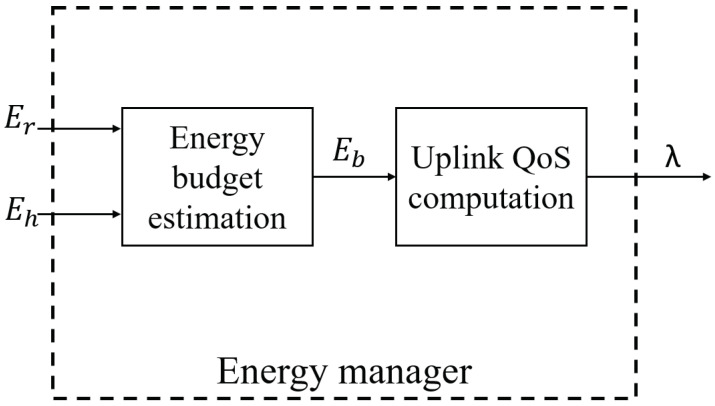
Energy manager: uplink Quality of Service (QoS) tuning procedure.

In this section a combination of the LoRa-WuR architecture with solar energy harvesting profile is considered. Therefore, the uplink QoS is tuned for each node according to the harvested energy with an energy manager. An energy manager is proposed and compared to two different energy managers. The proposed energy manager is called Redistribution of the Harvested Energy (RHE) that allows a small variation of the uplink QoS between days and nights. Figure 6 shows the procedure of the energy manager which consists in two blocks: the energy budget estimation and the uplink QoS computation. The energy manager is periodically executed at each slot $T_{s}$, the energy budget estimation block estimates the energy budget $E_{b}$ that corresponds to the energy that a node can consume in the next slot. Then, according to this $E_{b}$ and the used MAC protocol, the uplink QoS is calculated for each node.

### 5.1. Energy Harvesting Profile

**Figure 7:**
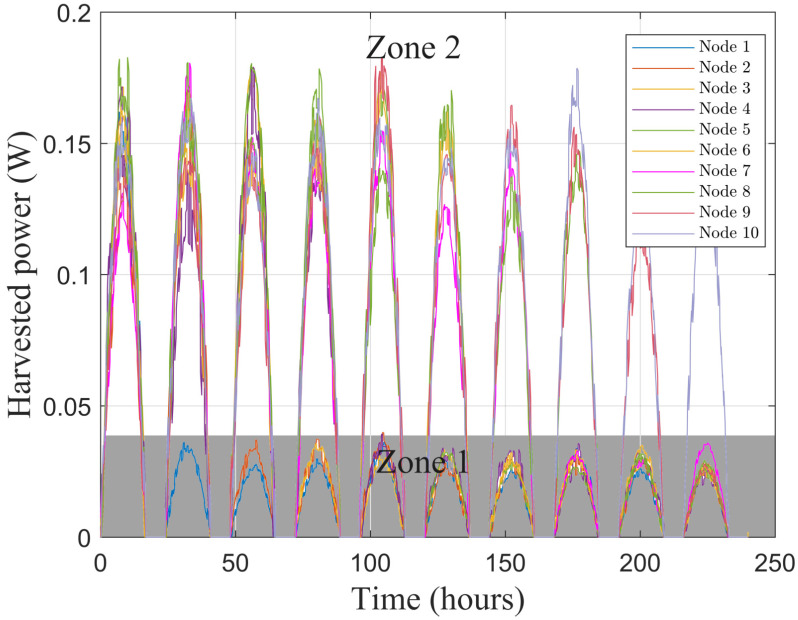
Harvested power for each of the 10 nodes during 10 days.

Outdoor solar daytime energy sources are considered in this study and power traces are generated by considering a solar panel area set to 30 cm${}^{2}$ for each node. As in real deployment some nodes will harvest less power than others, either because they are hidden from the solar source or due to a dysfunction of their solar panel. Therefore, the average power density is equal to 50 W/m${}^{2}$ for nodes that are in zone 2, whereas it is set to 10 W/m${}^{2}$ for nodes in zone 1 that harvest less energy. Figure 7 shows the harvested power traces generated for 10 nodes during 10 days using the algorithm proposed in Reference [28], with nights lasting for 8 h. To evaluate the influence of the ratio between the number of nodes in zone 1 and in zone 2, one node is moving each day from zone 2 to zone 1. The first day, all nodes are assumed to have the same average harvested power and are in zone 2. The second day, one node moves in zone 1 and has a reduced average harvested power and so on until the last day when nine nodes have a reduced average harvested power and only one node is still in zone 2 with a high average harvested power.

### 5.2. Energy Budget Estimation

To determine the uplink QoS of a node, an energy budget $E_{b}$ should be estimated. Therefore, three Energy Managers (EMs) are implemented to compare their performance: the first one is based on Fuzzyman [29], which is model-free; the second one is based on GRAPMAN [28], which allows a gradual tuning of the packet generation rate; and the novel one RHE is based on a redistribution of the harvested energy between days and nights. These three EMs are quite simple and incur few computations and, therefore, are well-adapted to wireless sensor networks.

The simulation was run in MATLAB for 10 days (simulation time) and super-capacitors of 15F are used to store energy as super-capacitors are more durable and also have higher power density when compared to rechargeable batteries [30].

Fuzzyman takes as inputs the residual energy denoted $E_{r}$ and the harvested energy denoted $E_{h}$. Fuzzyman is executed each slot $T_{s}$ fixed at 600 s. According to the harvested energy and the residual energy, $E_{b}$ is estimated for the next slot $T_{s}$ by considering five fuzzy sets with their membership functions [29]. Two sets concern the harvested energy called weak and strong, and three sets concern the residual energy and are called fail, empty and full. A minimum energy budget is fixed to 1 J, which is the energy required to allow a minimum uplink QoS corresponding to λ equal to 0.0103 packet/s, when using LoRa scheme, and it is equal to 0.0100 packet/s when using LoRa-WuR scheme.

GRAPMAN [28] gradually adapts the wake up interval (that is the reverse of the packet generation rate). At the beginning of each time slot $T_{s}$, GRAPMAN checks the wake up interval used at the previous slot. If the wake up interval prevents nodes from power failure, then it is decreased. Otherwise, it is increased. Thus, at each time slot, the wake up interval either stays the same, or is incremented by ±ΔTWI. ΔTWI is fixed to 1 s in our simulation, and the other parameters of the algorithm are used from Reference [28].

As Fuzzyman is a model-free that allows a high uplink QoS during the day and a low uplink QoS during the night, and GRAPMAN gradually tunes the uplink QoS without taking in consideration the ratio between days and nights, therefore, a novel energy budget estimation is proposed that allows a small fluctuation of the uplink QoS between days and nights. To tackle this issue and limit the uplink QoS variation, the idea of RHE is to store enough energy in the super-capacitor during periods of high harvested energy in order to reuse it during periods of energy scarcity (being at night for example). Unlike WVR -PM [30], RHE considers, at each slot, the harvested energy of the previous slot, whereas WVR-PM requires the estimation of the harvested energy of the next slot. At each slot kTs, RHE checks the harvested energy of the previous slot (k−1)Ts, if it is greater than certain threshold $E_{h}^{th}$ fixed at 10 J, the node uses $E_{b}\left(kTs\right)$ that is equal to:

(8) $$E_{b}\left(kTs\right)=\frac{TE}{TNE+TE}E_{h}\left((k-1)Ts\right),$$

with TE is the energy harvesting interval (when the energy harvesting is greater than $E_{h}^{th}$), and TNE is the non-energy harvesting interval (when the energy harvesting is lower than $E_{h}^{th}$) and it is equal to 10 h per day with the harvested profile described in Section 5.1. When the harvested energy is less than $E_{h}^{th}$, the node uses the stored energy in the super-capacitance. This technique allows a small variation of $E_{b}$ between days and nights.

### 5.3. Uplink QoS Computation

The uplink QoS expressed in terms of packet generation rate λ that will be calculated each $T_{s}$ once $E_{b}$ is estimated. $E_{b}$ corresponds to the energy that a node will consume during a time slot $T_{s}$; thus, to determine λ, the total energy consumption of a node should be derived and depends on the MAC protocol: LoRa scheme or LoRa-WuR in this study. A node using LoRa class A passes through different states, transmitting data (Tx), first wait window (W1w) before opening the first receive window (Rx1w), a second wait window (W2w) before opening the second receive window (Rx2w), and, finally, come back to sleep state. The description of these states and their duration and power consumption are illustrated in Table 3. The duration $T_{tx}$ (that is equal to the Time on Air), $T_{Rx1w}$, $T_{W2w}$, and $T_{Rx2w}$ depend on the used data rate [31] that is given in config. 1 of Table 2.

The average uplink QoS when using LoRa scheme denoted $\lambda_{LoRa}$ can be expressed as:

(9) $$\lambda_{LoRa}=\frac{E_{b}-P_{sleep}T_{s}}{\left(T_{Tx}P_{Tx}+T_{W1w}P_{W1w}+T_{Rx1w}P_{Rx1w}+T_{W2w}P_{W2w}+T_{Rx2w}P_{Rx2w}-P_{sleep}T_{act}\right)T_{s}},$$

with $T_{act}$ the duration of all states related to the node activities except sleep state, and it is expressed as:

(10) $$T_{act}=T_{Tx}+T_{W1w}+T_{Rx1w}+T_{W2w}+T_{Rx2w}.$$

When using LoRa-WuR, as an OCH will forward a CMD to a targeted node, then its power consumption will be increased. To compensate this increase of power and consume the same as when using the traditional LoRa scheme, the node using LoRa-WuR will reduce its uplink QoS; thus, the uplink QoS when using LoRa-WuR denoted $\lambda_{LoRa-WuR}$ is expressed as:

(11) $$\lambda_{LoRa-WuR}=\frac{E_{b}-P_{sleep}T_{s}-P_{idle}^{wur}T_{s}}{\left(P_{act}T_{act}+e_{cmd}^{wutx}+\left(N-1\right)e_{cmd}^{wurx}-\left(N-1\right)P_{idle}^{wur}T_{wub}-P_{sleep}T_{act}^{LoRa-WuR}\right)T_{s}},$$

with $T_{act}^{LoRa-WuR}$ the duration of all states related to the node activities including the duration of forwarding the CMD as form of a WUB and it is equal to:

(12) $$T_{act}^{LoRa-WuR}=T_{act}+l_{wur}.$$

### 5.4. Downlink QoS and Downlink Latency Evaluation

**Figure 8:**
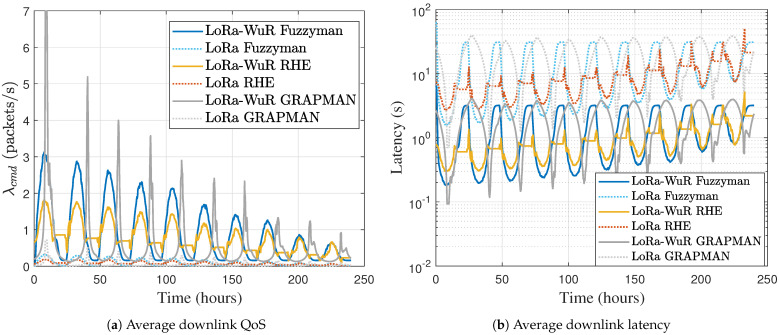
Average downlink QoS and average downlink latency during 10 days.

The downlink latency depends on the downlink QoS (i.e., received CMD rate), and it can be calculated for both LoRa and LoRa-WuR schemes by (2) and (4). Further, in the considered scheme, the downlink QoS directly depends on the uplink QoS, following (3) and (5). And the uplink QoS (i.e., packet generation rate) is finally calculated by (9) and (11) according to the used MAC scheme. Figure 8a shows the average downlink QoS during 10 days, with both the traditional LoRa (dashed line) and LoRa-WuR (continued line) schemes, and with the three different EMs. It appears that when there is light, Fuzzyman achieves a downlink QoS between GRAPMAN and RHE, and, by night, it achieves the minimum downlink QoS. GRAPMAN can achieve the highest downlink QoS but in a short time horizon when there is light, and in the night the downlink QoS achieves its minimum, like Fuzzyman. RHE shows a minimum fluctuation of the downlink QoS between night and day. When comparing LoRa and LoRa-WuR schemes(the dashed line compared to the continued line), it is shown that with LoRa-WuR the downlink QoS is 10 times higher than when using the traditional LoRa scheme, as all nodes contribute to forward the CMD in the SR. The average downlink QoS and its variance according to the used EM, and with both the traditional LoRa scheme $\lambda_{cmd}^{LoRa}$ and LoRa-WuR $\lambda_{cmd}^{LoRa-WuR}$, are summarized in Table 4.

The downlink latency with both schemes LoRa and LoRa-WuR are calculated by (2) and (4). Figure 8b shows the average downlink latency during the 10 days with both the traditional LoRa (dashed line) and LoRa-WuR (continued line) schemes, and with the three EMs. It can be seen that regardless of the used EM, the downlink latency is reduced when using LoRa-WuR scheme compared to the traditional LoRa scheme. RHE allows to have the lowest fluctuation on the downlink latency, so at any time, the ED can receive the CMD from the gateway with roughly the same downlink latency. This is contrary to Fuzzyman and GRAPMAN, where the downlink latency depends on the zone where the ED is present (harvest less or more energy). Table 4 summarizes the average downlink latency with both schemes and with the three EMs. The downlink latency is reduced in average of a factor 9.5 (corresponding to a reduction by almost 90%) with LoRa-WuR scheme compared to the traditional LoRa scheme.

## 6. Conclusions

This paper presents a MAC protocol for heterogeneous architecture combining LoRa for long range communication and wake-up radio (WuR) for short range communication (LoRa-WuR). Nodes are equipped with solar panel for harvesting energy, and a novel energy manager is proposed allowing an adaptive tuning of the quality of service for each node. Moreover, as, in a real deployment, nodes that harvest energy are subject to different perturbations, e.g., solar panels are hidden from the solar source, then two zones are considered in which nodes harvest a high or a low power. Results show that even if nodes harvest less energy, the downlink latency using LoRa-WuR can be reduced compared to the traditional LoRa scheme. It can be reduced by almost 90% for a cluster of 10 nodes. Thanks to the protocol LoRa-WuR, nodes are cooperative with each other and each node can contribute to forward commands received from the gateway to another node. This technique resolves the traditional bottleneck of LoRa protocol in which the gateway has not a full control of the network and has a long downlink latency. Furthermore, the more the nodes are present in the cluster, the more the downlink latency is reduced with LoRa-WuR (the maximum number of nodes that can be used in a cluster is, however, naturally limited by the short range of the WuR).

## Figures and Tables

**Table 1:** Measured values from microbenchmark.

Parameter	Value	Parameter	Value
$e_{cmd}^{wutx}$	2.19 mJ	$P_{idle}^{wur}$	1.83μW
$e_{cmd}^{wurx}$	4.5μJ	$R_{WUB}$	1 kbps
$e_{cmd}^{L}$	92.52 mJ	$L_{WUB}$	2 bytes

**Table 2:** Setup used for LoRa.

Config. 1		Config. 2	
Parameter	Value	Parameter	Value
SF	6	SF	12
BW	500 kHz	BW	125 kHz
CR	$\frac{4}{5}$	CR	$\frac{4}{8}$
$R_{b}$	37.5 kbps	$R_{b}$	0.183 kbps
Payload	5 bytes	Payload	5 bytes
$l_{cmd}$	5.6 ms	$l_{cmd}$	1.09 s

**Table 3:** Parameters description and their values.

Parameter	Description	Value	Unit
$T_{Tx}$	Time to transmit a packet (Time on Air)	5.6	ms
$P_{Tx}$	Power consumption when transmitting with LoRa	273.9	mW
$T_{W1w}$	Duration of the first wait window	983.3	ms
$P_{W1w}$	Power consumption during the first wait window	89.1	mW
$T_{Rx1w}$	Duration of the first receive window	5.6	ms
$P_{Rx1w}$	Power consumption of the first receive window	115.5	mW
$T_{W2w}$	Duration of the second wait window	978.1	ms
$P_{W2w}$	Power consumption during the second wait window	89.1	mW
$T_{Rx2w}$	Duration of the second receive window	33	ms
$P_{Rx2w}$	Power consumption of the second receive window	115.5	mW
$P_{sleep}$	Power consumption during sleep state	148.5 × $10^{-3}$	mW

**Table 4:** Evaluation of both average downlink QoS and average downlink latency for Fuzzyman, Harvested Energy (RHE), and GRAPMAN.

	EMs		Fuzzyman	RHE	GRAPMAN
Metrics					
CMD rate average (packet/s)		${\bar{\lambda }}_{cmd}^{LoRa}$	0.090	0.077	0.056
		${\bar{\lambda }}_{cmd}^{LoRa-WuR}$	0.883	0.750	0.548
CMD rate variance (packet/s)		$\bar{\sigma }\left(\lambda_{cmd}^{LoRa}\right)$	0.007	0.002	0.008
		$\bar{\sigma }\left(\lambda_{cmd}^{LoRa-WuR}\right)$	0.632	0.154	0.729
Downlink latency (s)		$L_{LoRa}$	13.32	8.78	19.56
		$L_{LoRa-WuR}$	1.39	0.92	2.03

## Data Availability

The data presented in this study are available on request from the corresponding author.
